# Ancestral chromosomal signatures of Paenungulata (Afroteria) reveal the karyotype of Amazonian manatee (*Trichechus inunguis*, Sirenia: Trichechidae) as the oldest among American manatees

**DOI:** 10.1186/s12864-023-09129-3

**Published:** 2023-01-24

**Authors:** Flávia dos Santos Tavares, Willam Oliveira da Silva, Malcolm Andrew Ferguson-Smith, Alex Garcia Cavalleiro de Macedo Klautau, Jairo Moura Oliveira, Angélica Lúcia Figueiredo Rodrigues, Gabriel Melo-Santos, Julio Cesar Pieczarka, Cleusa Yoshiko Nagamachi, Renata Coelho Rodrigues Noronha

**Affiliations:** 1 Laboratório de Citogenética, Centro de Estudos Avançados da Biodiversidade, Instituto de Ciências Biológicas, Universidade Federal Do Pará (UFPA), Pará Belém, Brazil; 2 Cambridge Resource Centre for Comparative Genomics, Department of Veterinary Medicine, University of Cambridge, Cambridge, UK; 3 Centro Nacional de Pesquisa e Conservação de Mamíferos Aquáticos, Instituto Chico Mendes de Conservação da Biodiversidade, Pará Belém, Brazil; 4 Zoological Park of Santarém – Universidade da Amazônia (ZOOUNAMA), Pará Santarém, Brazil; 5 Instituto de Biologia e Conservação de Mamíferos Aquáticos da Amazônia, Universidade Federal Rural da Amazônia (UFRA), Pará Belém, Brazil; 6 Secretaria de Educação Do Estado Do Pará (SEDUC-PA), Belém, Brazil; 7 Laboratório de Ecologia Marinha e Conservação, Universidade Federal do Rio de Janeiro, Rio de Janeiro Rio de Janeiro, Brazil; 8 Laboratório de Ecologia de Aves e Comportamento Animal, Universidade Estadual do Rio de Janeiro, Rio de Janeiro Rio de Janeiro, Brazil

**Keywords:** Chromosomal evolution, Sirenians, Ancestral karyotype, ZOO-FISH

## Abstract

**Background:**

Chromosomal painting in manatees has clarified questions about the rapid evolution of sirenians within the Paenungulata clade. Further cytogenetic studies in Afrotherian species may provide information about their evolutionary dynamics, revealing important insights into the ancestral karyotype in the clade representatives. The karyotype of *Trichechus inunguis* (TIN, Amazonian manatee) was investigated by chromosome painting, using probes from *Trichechus manatus latirostris* (TML, Florida manatee) to analyze the homeologies between these sirenians.

**Results:**

A high similarity was found between these species, with 31 homologous segments in TIN, nineteen of which are whole autosomes, besides the X and Y sex chromosomes. Four chromosomes from TML (4, 6, 8, and 9) resulted in two hybridization signals, totaling eight acrocentrics in the TIN karyotype. This study confirmed in TIN the chromosomal associations of *Homo sapiens* (HSA) shared in Afrotheria, such as the 5/21 synteny, and in the Paenungulata clade with the syntenies HSA 2/3, 8/22, and 18/19, in addition to the absence of HSA 4/8 common in eutherian ancestral karyotype (EAK).

**Conclusions:**

TIN shares more conserved chromosomal signals with the Paenungulata Ancestral Karyotype (APK, 2n = 58) than *Procavia capensis* (Hyracoidea), *Loxodonta africana* (Proboscidea) and TML (Sirenia), where TML presents less conserved signals with APK, demonstrating that its karyotype is the most derived among the representatives of Paenungulata. The chromosomal changes that evolved from APK to the *T. manatus* and *T. inunguis* karyotypes (7 and 4 changes, respectively) are more substantial within the *Trichechus* genus compared to other paenungulates. Among these species, *T. inunguis* presents conserved traits of APK in the American manatee genus. Consequently, the karyotype of *T. manatus* is more derived than that of *T. inunguis*.

## Background

Paenungulata (Afrotheria) includes the orders Proboscidea ILLIGER 1811, Hyracoidea HUXLEY 1869, and Sirenia ILLIGER 1811, established by morphological, genomic and cytogenetic evidence, despite the controversial phylogenetic position between these orders [1–5].

The order Sirenia are exclusively aquatic herbivorous mammals, composed of two families, Dugongidae (dugongs) and Trichechidae (manatees), that probably diverged in the early Eocene, 56 million years ago (myr) [6–10]. The Trichechidae family is divided into Miosireninae (extinct) and Trichechinae (current manatees) subfamilies. Three species of the *Trichechus* genus represent the current manatees, *Trichechus manatus* LINNAEUS 1758 (West Indian manatee), *Trichechus senegalensis* LINK 1795 (African manatee) and *Trichechus inunguis* NATTERER 1883 (Amazonian manatee). The taxon is distributed in the tropical and subtropical regions of the Atlantic Ocean: *T. manatus* lives in the Atlantic coastal region of the Americas, *T. senegalensis* in the rivers and coastal areas of western Africa and *T. inunguis* is endemic to Amazonian rivers [11].

Morphological data established the first phylogenetic relationships of trichequid representatives, suggesting that the first manatees have ancestry from estuarine regions and freshwater environments in South America [7, 12, 13]. Fossil analysis, through studies of tooth morphology, inferred that *Ribodon limbatus* AMEGHINO 1883 is an ancestor of the genus *Trichechus* [7, 12, 14]. Domning [7, 12] proposed that *T. inunguis* is the most recent species among the representatives of *Trichechus* based on morphology and paleogeographic history.

The mitochondrial gene data described by Vianna et al. [15] strengthened the phylogenetic relationship between *T. manatus* and *T. senegalensis*, corroborating the morphological phylogenetic interpretations [7, 12]. However, *Cyt b* genes in *T. inunguis* showed a lower degree of sequence changes concerning *T. manatus* and *T. senegalensis*, indicating the sequence in *T. inunguis* as the most conserved among *Trichechus*, although the study concluded that *T. inunguis* would be the most recent species. De Souza et al. [16] analyzed the mitochondrial genomes of *Trichechus* representatives and proposed the time of evolutionary divergence between the species at 6.5 myr. In addition, the study presented *T. senegalensis* as the oldest species among the *Trichechus*. It established a closer relationship between *T. manatus* and *T. inunguis*, mainly considering the divergence time at 1.34 myr between the two species. These divergence times are very short, considering the significant phenotypic differences between these species [11, 16]. From a morphological perspective, it is possible to confirm the proximity between *T. manatus* and *T. senegalensis* due to the similarity in habitat and niches of these species, which contribute to the preservation of typical phenotypes in marine manatees. However, despite the genomic data by Vianna et al. [15] reinforcing this proximity of *T. manatus* and *T. senegalensis*, the findings in *T. inunguis* were controversial in relation to the phylogenetic interpretations already described for the species. The similarity of mitogenomes between *T. manatus* and *T. inunguis* described by De Souza et al. [16] proposes, for the first time, a different phylogenetic interpretation for the group.

Chromosome painting has been effective in clarifying information about evolutionary aspects of mammals and assessing karyotypic and phylogenetic ancestry, as well as evolutionary divergence between taxonomic groups [17, 18]. Cytogenetic analyzes available in the literature for *Trichechus* showed the established diploid number (2n) and autosomal fundamental number (FN) for *T. inunguis* as 2n = 56/FN = 82 [19–22] and 2n = 48/FN = 92 for *T. manatus* [22–24]. This variation in karyotypes is remarkable, with a difference of four Robertsonian rearrangements [19] between *T. manatus* and *T. inunguis*, considering the short divergence time (1.34 myr) between these species. More recent data from Noronha et al. [22] and De Oliveira et al. [20], based on karyotypic analysis, demonstrated chromosomes rearrangements and the natural occurrence of hybrids from reproduction between *T. inunguis* and *T. manatus* or different generations (F1, F2). Cytogenetic data for *T. senegalensis* have not yet been described.

Cytogenetic analyzes of the African elephant (*Loxodonta africana*, 2n = 56), Florida manatee (*Trichechus manatus latirostris*, 2n = 48), and hyrax (*Procavia capensis*, 2n = 54), by chromosome painting and comparative analysis with *Homo sapiens* (HSA), show chromosomal signatures that validate the ancestral karyotype of Eutheria (EAK), with HSA 3/21, 7/16, 12/22, 14/15, and 16/19 syntenies, in addition to consolidating the Paenungulata clade with HSA 2/3, 8/22, and 18/19 syntenies [2, 18, 25]. Furthermore, Pardini et al. [2], using chromosome painting in *T. m. latirostris* (Sirenia), *L. africana* (Proboscidea), and *P. capensis* (Hyracoidea), established the karyotypic differences between these species and confirmed 11 synapomorphies that characterize the Paenungulata clade, in addition to establishing the ancestral karyotype (APK, 2n = 58).

Therefore, the verification and number of chromosomal changes that have occurred during the divergence of *T. manatus* and *T. inunguis* could help to elucidate the phylogenetic interpretations described for the genus *Trichechus*. Here, data on chromosome painting in *Trichechus inunguis*, and the evolutionary aspects that differentiate the manatees *T. manatus* and *T. inunguis* and their phylogenetic relationships, are shown for the first time on a comparative chromosomal analysis with other representatives of the Paenungulata clade available from the published data.

## Results

The karyotype of *Trichechus inunguis* (TIN) presents 2n = 56, FN = 92, and an XX/XY sex chromosome system. Of the autosome chromosomes, 19 pairs are bi-armed and 8 one-armed; the X is submetacentric, and the Y is acrocentric.

**Fig. 1:**
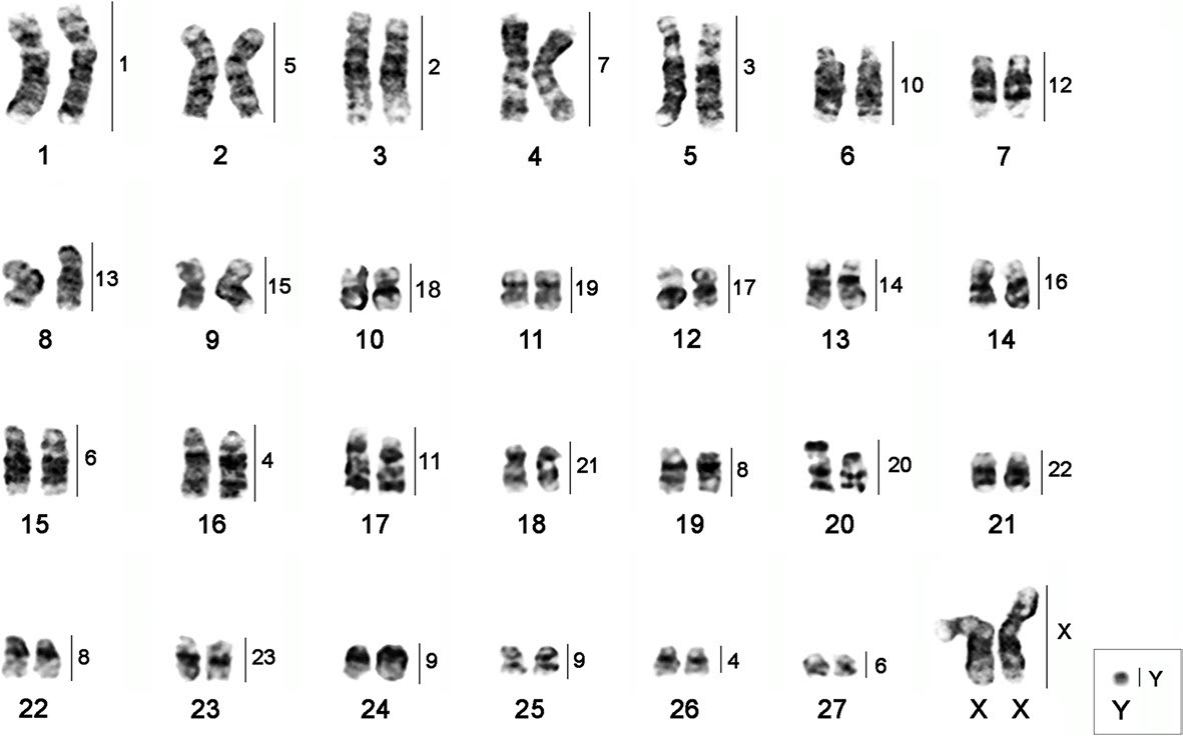
G-banded karyotype of *Trichechus inunguis* (2n = 56, FN = 92) [22], with chromosomal mapping plotted from hybridizations with whole chromosome probes from *Trichechus manatus latirostris (2n = 48, FN = 92)*

**Fig. 2:**
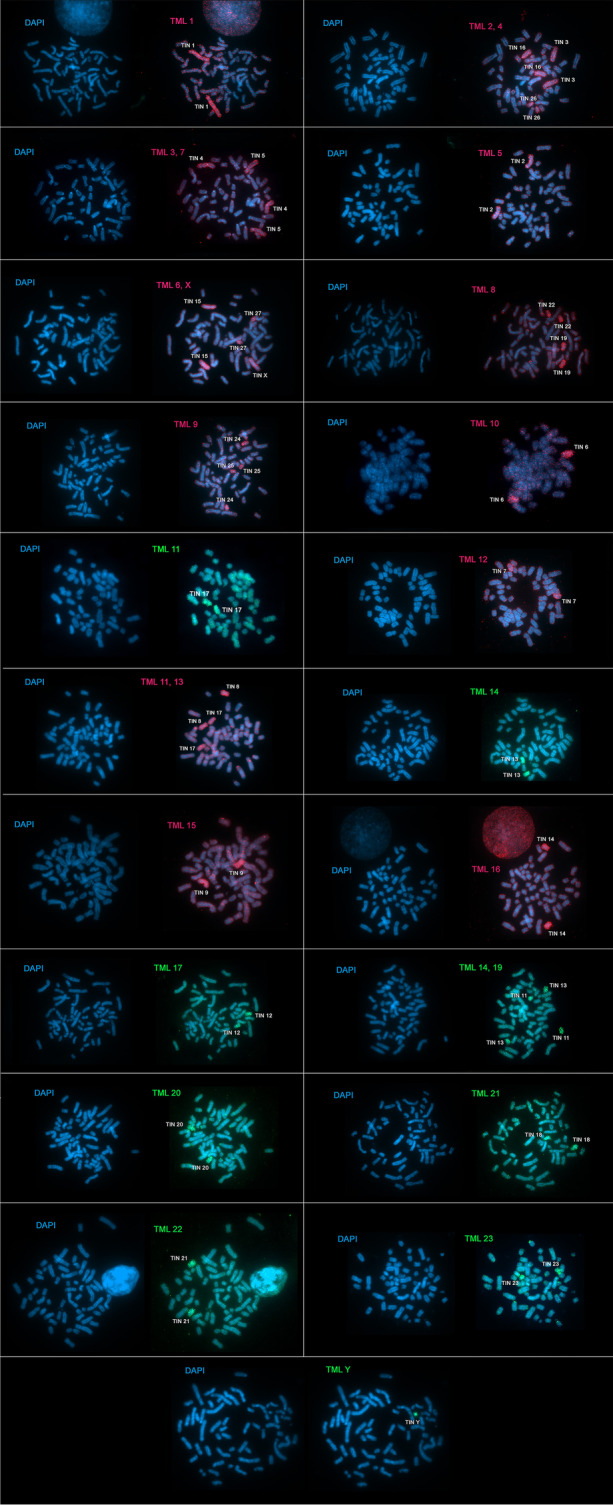
FISH with probes from *Trichechus manatus latirostris* (TML) in *Trichechus inunguis* (TIN). The probes are shown in red (Cy3) or green (FITC). Chromosomes counterstaining in blue (DAPI)

Hybridization of *T. m. latirostris* (TML) probes in TIN demonstrates 31 homeologous segments. Of these, we identified nineteen (TML 1, 2, 3, 5, 7, 10, 11, 12, 13, 14, 15, 16, 17, 18, 19, 20, 21, 22, and 23) that hybridized to a single autosomal chromosomes of TIN (TIN 1, 3, 5, 2, 4, 6, 17, 7, 8, 13, 9, 14, 12, 10, 11, 20, 18, 21, and 23, respectively), in addition to the TML X and Y in TIN X and Y, respectively; four TML chromosomes showed two hybridization signals: TML 4 (TIN 16 and 26), TML 6 (TIN 15 and 27), TML 8 (TIN 19 and 22), and TML 9 (TIN 24 and 25) (Fig. 1 and Fig. 2; Table 1).

**Table 1:** FISH results in *Trichechus inunguis* (TIN, 2n = 56) from *T. manatus latirostris* (TML, 2n = 48) whole chromosome probes

TML	TIN
1	1
2, 4	3, 16, 26
3, 7	4, 5
5	2
6, X	15, 27, X
8	19, 22
9	24, 25
10	6
11	17
11, 13	8, 17
12	7
14	13
15	9
16	14
17	12
18	10
14, 19	11, 13
20	20
20	20
17, 21	18
22	21
23	23
Y	Y

Additionally, when comparing by G band and chromosome painting the TML, TIN, *Loxodonta africana* (LAF) and *Procavia capensis* (PCA) species, we observed that TIN 1 underwent a pericentric inversion when compared to TML 1; and, TIN 2 (TML 5) and TIN 4 (TML 7) underwent centromere inversion/repositioning when compared to LAF (LAF 5 and LAF 17; LAF 4) and PCA (PCA 4; PCA 3), respectively [2, 22].

## Discussion

### Comparative analysis between TIN and TML

The comparative analysis between TIN and TML was proposed based on the results of Kellogg et al. [25], with hybridizations of *Homo sapiens* (HSA) probes in TML and the effects of hybridizations with TML probes in TIN of the present study. Therefore, the data found in TML were used as an intermediary to infer the chromosomal associations of HSA in TIN due to the high degree of genome similarity observed in the hybridizations between these species.

**Fig. 3:**
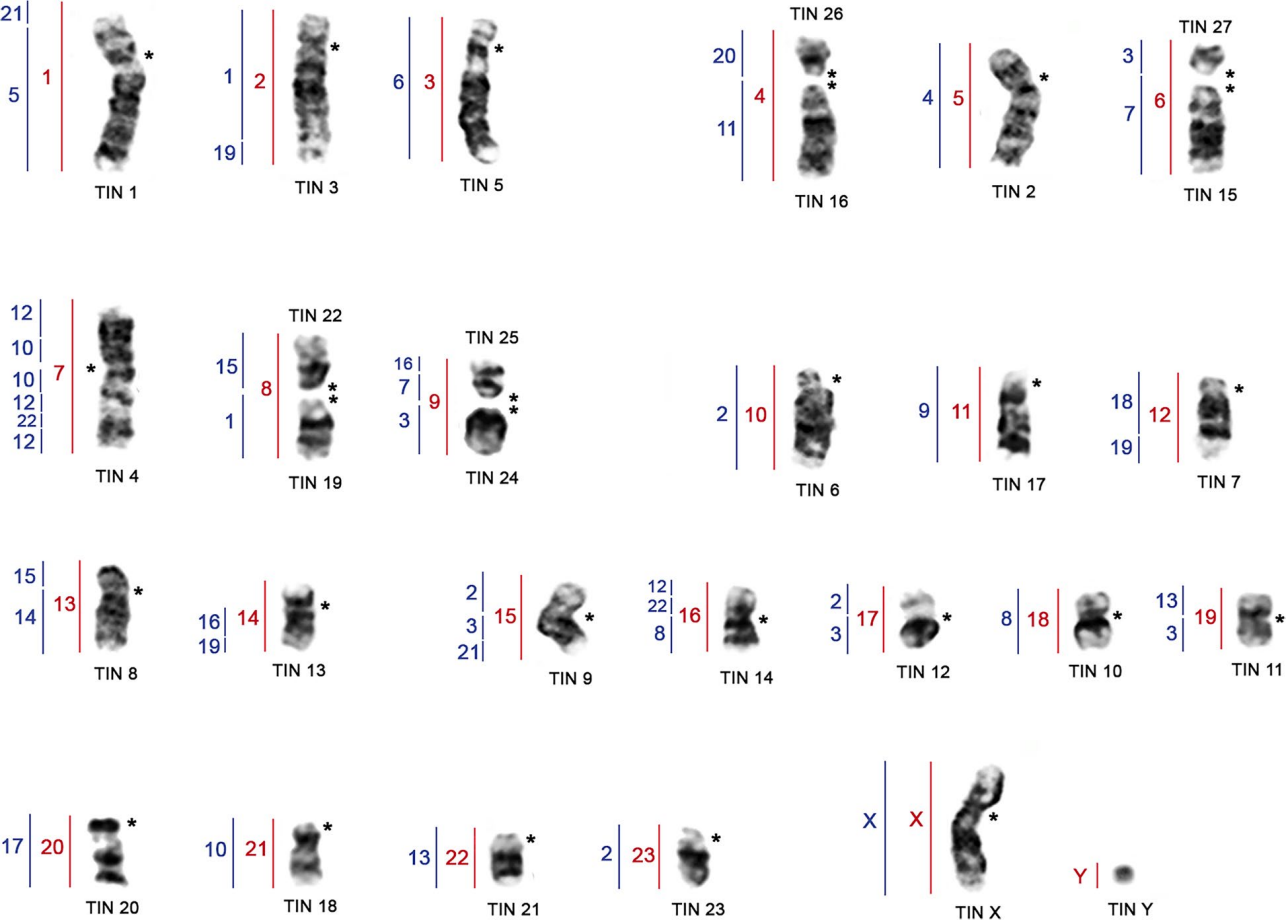
Comparative analysis by chromosome painting between *T. m. latirostris* (TML; red bar) and *T. inunguis* (TIN) (present study) and *Homo sapiens* (HSA; blue bar) with TML [25]. (*) represent centromeric regions

Common associations were observed in the ancestral Eutheria karyotype (AEK) with the HSA syntenies 3/21 (TIN 9), 7/16 (TIN 25), 12/22 in two blocks (TIN 4 and TIN 14), 14/15 (TIN 8), and 16/19 (TIN 13); and the association HSA 5/21 (TIN 1) for the Afrotheria clade, despite the HSA 5/21 gap in the karyotype of *Procavia capensis* [2]. Paenungulata ancestral karyotype (APK) associations were also found in *T. inunguis*, with HSA 2/3 syntenies in two blocks (TIN 9 and TIN 12), 18/19 (TIN 7), 8/22 (TIN 14) (see Fig. 3 and Table 3). HSA 4/8 synteny is common in AEK and has been detected in Afroinsectiphilia (African insectivores) [26–29]. However, it was not observed in *T. inunguis*, as well as in *L. africana*, *T. m. latirostris*, and *P. capensis* [2, 25, 30], reinforcing that this association was lost in the representatives of Paenungulata.

### Comparative analyzes of the Paenungulata Ancestral Karyotype (APK) in Amazonian manatee

Cytogenetic studies on sirenians are still restricted to manatees *T. manatus* and *T. inunguis* [2, 19, 21, 23–25]. The two species have strikingly different karyotypes (*T. inunguis* 2n = 56; *T. manatus* 2n = 48), with a difference of four Robertsonian translocations and one pericentric inversion [22].

**Fig. 4:**
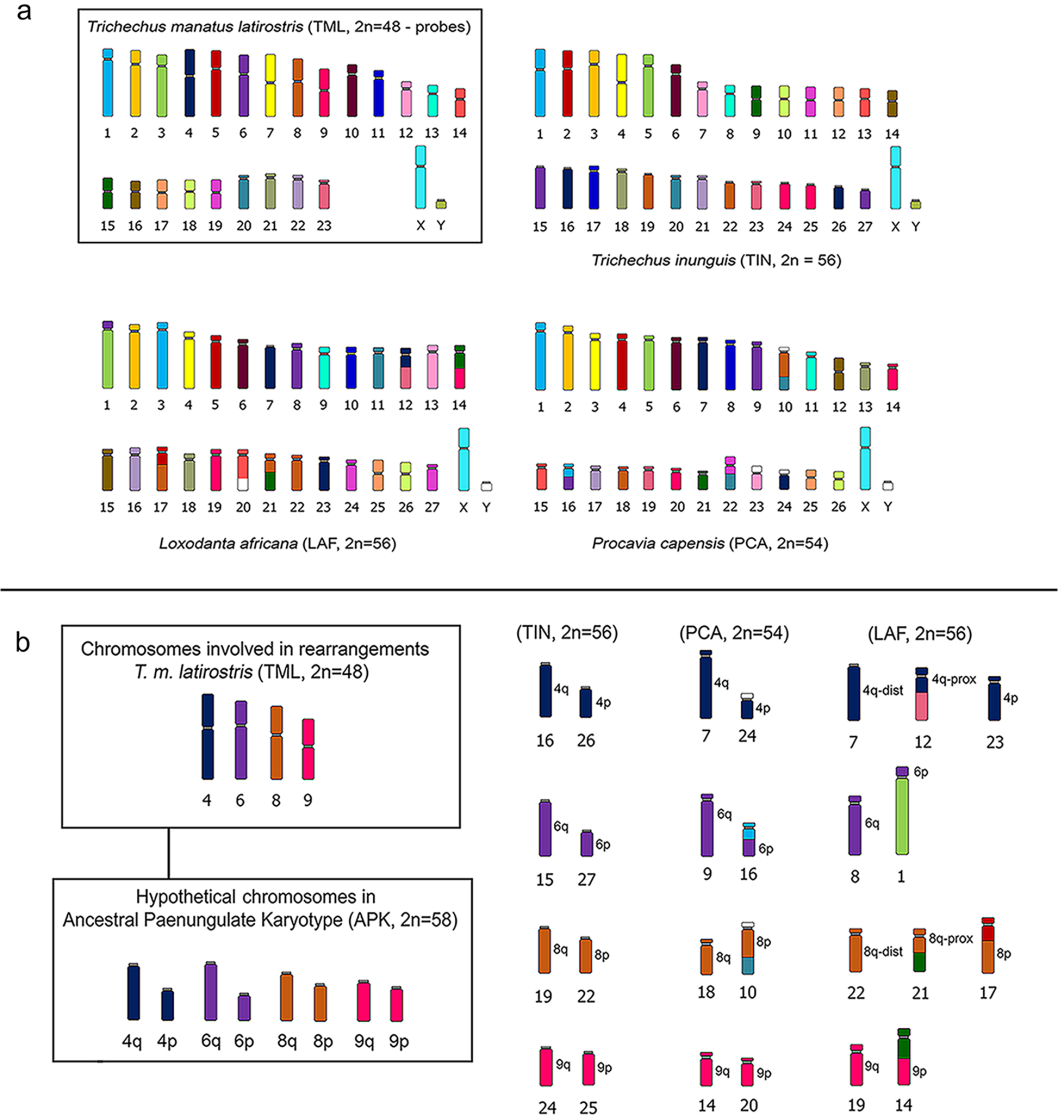
a) Representative idiograms of chromosome painting in *Trichechus inunguis* (TIN, 2n = 56), *Loxodonta africana* (LAF, 2n = 56), and *Procavia capensis* (PCA, 2n = 54) with *T. m. latirostris* (TML, 2n = 48); b) Chromosomal changes involving TML chromosomes 4, 6, 8, and 9 and the possible corresponding chromosomes of APK, 2n = 58 in TIN, PCA, and LAF

Comparative analysis by chromosome painting with TML probes between the TIN karyotype and the paenungulate representatives (*Trichechus manatus latirostris* – TML, *Loxodonta Africana* – LAF and *Procavia capensis* – PCA) corroborate the data found by Pardini et al. [2] who described the Ancestral Karyotype of Paenungulata (APK) (Table 3 and Fig. 4a and b). Comparative analysis by chromosome painting showed that the TIN (2n = 56) and TML (2n = 48) karyotypes differ by 4 fusion/fission events between 8 acrocentric pairs in TIN and 4 submetacentric pairs in TML (Fig. 1). The alterations detected in the TIN karyotype involving the TML chromosomes 4, 6, 8, and 9 also occurred in PCA and LAF, which are fragmented into two to three blocks in these karyotypes, respectively (Fig. 4b; Table 2) [2]. Considering the four Robertsonian rearrangements in TIN (Based on TML chromosomes 4, 6, 8 and 9 hybridization) we suggest that the TIN karyotype is more ancestral than the TML karyotype, since the latter is more similar to the Ancestral Paenungulate Karyotype (APK).

**Table 2:** Rearrangements of chromosomes 4, 6, 8, and 9 of *Trichechus manatus latirostris* (TML) in representatives of Paenungulata: *Loxodonta africana* (LAF) and *Procavia capensis* (PCA), data from Pardini et al. [2] and from present study on *Trichechus inunguis* (TIN)

Species	2n	TML 4	TML 6	TML 8	TML 9
**PROBOSCIDEA**					
*Loxodonta africana*	56	LAF 23 (TML 4p) LAF 12p-qprox (TML 4q-prox) LAF 7 (TML 4q-dist)	LAF 1p (TML 6p) LAF 8 (TML 6q)	LAF 17q-dist (TML 8p) LAF 21pq-prox (TML 8q-prox) LAF 22 (TML 8q-dist)	LAF 14q-dist (TML 9p) LAF 19 (TML 9q)
**HYRACOIDEA**					
*Procavia capensis*	54	PCA 24 (TML 4p) PCA 7 (TML 4q)	PCA 16q-dist (TML 6p) PCA 9 (TML 6q)	PCA 10pq-prox (TML 8p) PCA 18 (TML 8q)	PCA 20 (TML 9p) PCA 14 (TML 9q)
**SIRENIA**					
*Trichechus inunguis*	56	TIN 26 (TML 4p) TIN 16 (TML 4q)	TIN 27 (TML 6p) TIN 15 (TML 6q)	TIN 22 (TML 8p) TIN 19 (TML 8q)	TIN 25 (TML 9p) TIN 24 (TML 9q)

Our data corroborate those of Pardini et al. [2] and confirms that the TIN karyotype maintained the 11 synapomorphies proposed in the paenungulate representatives TML, LAF, and PCA, validated by the karyotype of the outgroup, aardvark (*Orycteropus afer*, 2n = 20). Furthermore, the study showed that the Ancestral Paenungulata Karyotype (APK) would consist of 2n = 58 chromosomes, validated by the karyotype of the outgroup, aardvark (2n = 20). Comparative analyzes from the APK indicate that *L. africana* (2n = 56) underwent 5 fusions, 4 fissions, and 1 inversion/centromere repositioning on chromosome 3 (LAF 3) to constitute the current karyotype; *P. capensis* (2n = 54) underwent 4 fusions and 2 fissions; *T. m. latirostris* underwent 5 fusions and 2 inversion/centromere repositioning (TML 5 and 7) [2]. From the same perspective of analysis by Pardini et al. [2], the analysis from this present study showed that *T. inunguis* showed a karyotype modification of 1 fusion (in TIN 9), 1 pericentric inversion (TIN 1) (by Noronha et al. [22]) and 2 inversion/centromere repositioning (TIN 2 and 4), indicating a more conserved karyotype with APK than other paenungulates (Table 3).

**Table 3:** Ancestral Paenungulata Karyotype (APK) with 2n = 58, XY [2]. APK homologies in representatives of Paenungulata (*L. africana* – LAF; *T. m. latirostris* – TML; *P. capensis* – PCA) and *T. inunguis* (TIN) data from the present study, considering *Orycteropus afer* (OAF) and *Homo sapiens* (HSA) as an outgroup. Chromosome painting data from Pardini et al. [2] and FISH data with TML probes in TIN. The question marks (?) are regions not yet resolved by the chromosomal painting. The abbreviation *inv.* indicates pericentric inversion and *inv/cr* indicates in which chromosomes there was inversion/centromeric repositioning

APK	OAF	LAF	TML	PCA	TIN	HSA
1	2qhi	3 *inv/cr*	1	1,16p?	1 *inv*	5/21
2	3qcd	2	2	2	3	1/19
3	3p	1q	3	5	5	6
4	1qhi	5, 17	5 *inv/cr*	4	2 *inv/cr*	4
5	4q	4	7 *inv/cr*	3	4 *inv/cr*	10p/12/22q-dist
6	6qa	6	10	6	6	2q
7	4p	7, 12	4q	7	16	11
8	7q	10	11	8	17	9
9	5p	8	6q	9	15	7
10	5qbc	9	13	11	8	14
11	7p	15	16	12	14	8q
12	8q	18	21	13	18	10q
13	1pbc	20	14	15	13	16q
14	1qa	16	22	17	21	13
15	3qab	21, 22	8q	18	19	1
16	1qc	12	23	19	23	2pq-prox
17	6qbc	14	9p	20	25	7/16p
18	1qe	14	15p	21	9	2pq-prox
19	2qa	19	9q	14	24	3
20	8p	11	20	10, 22	20	17
21	5qa	17	8p	10	22	15
22	2qfg	21	15q	?	9	3/21
23	2q	1p	6p	16	27	3
24	1pa + 6p	13	12	23	7	19q/18
25	1qf + 9q	25	17	25	12	8p/22q-prox
26	2p	23	4p	24q	26	20
27	1qb + 2qd	26	18	26	10	3/13q
28	1qd + 2qb (c)	27 (24)	19	22	11	2pq-prox/3

### The rapid dissemination of the *Trichechus* genus

The paleoenvironmental dynamics that occurred in South America during the Cenozoic were responsible for the diversification and distribution of the first representatives of the genus *Trichechus* [12]. During the formation of the Amazon basin, the Andean elevation generated different landscapes that benefited the diversity of the South American biota [31–33]. The discovery of the *Potamosiren* fossil links the first manatees to the estuarine and freshwater environments of South America [7, 12]. The constant marine transgressions that occurred on the continent in the Neogene (Miocene and Pliocene) may have caused the reintroduction of sirenians into fresh waters, as the broad community of sirenians of the Tertiary was marine in origin [6, 9, 13, 32–34].

The first *Trichechus* diverged by allopatry in marine and freshwater environments. Within the Amazon basin, the *Trichechus* genus modified its diet; the high production of macrophytes and other abrasive grasses selected the first isolated *Trichechus*; outside the Amazon basin, marine *Trichechus* took different routes and diversified; *Trichechus senegalensis*, in coastal regions and rivers of tropical West Africa; and *Trichechus manatus*, in the coastal area of the American continents [12]. Fossil data for these manatees are still too scarce to suggest past distribution. However, the diversity of *Trichechus manatus* in the lineage-subspecies *T. manatus bakerorum* (extinct), *T. manatus latirostris* (Florida manatee), *T. manatus manatus* (Antillean manatee), and *T. manatus manatus* (Brazilian *T. manatus*) along the American Atlantic coast support a state of rapid diversification within the genus *Trichechus*, validated by morphological, genomic and cytogenetic characteristics [14, 15, 35–38].

Although phylogenetic positions are still controversial among extant *Trichechus* [12, 15, 16, 39], genomic data have estimated the time of evolutionary divergence between these species. The analysis by Cantanhede et al. [36] with D-loop between *T. manatus* and *T. inunguis* estimated the time of evolutionary divergence from 3.1 to 0.65 myr, while the complete mitochondrial genomes analyzed by De Souza et al. [16] showed an evolutionary divergence between *T. manatus* and *T. inunguis* of 1.34 myr. The short time of divergence between these species can be seen in our data due to the high chromosomic similarity found in the present study, which can also support the existence of natural hybridization between *T. manatus* and *T. inunguis* in the Amazon estuary [20, 22]. The estimated rate of chromosomal changes in Paenungulata is considered slow to moderate (0.09 – 0.16 changes per 1 million years – changes/myr) compared to other mammalian groups [2]. The chromosomal changes for the paenungulate of the orders Hyracoidea (*P. capensis* – 2n = 54) and Proboscidea (*L. africana* – 2n = 56) show a difference of 6 to 9 changes in APK, respectively, given that the evolutionary divergence of these taxa has been approximately 56 myr [1, 40]. In addition, other known representatives of Hyracoidea (*Dendrohyrax arboreus*, 2n = 54; *Heterohyrax hrucei*: 2n = 54) and Proboscidea (*Elephas maximus*, 2n = 56) still maintain a conserved diploid number [41, 42]. However, the difference of four Robertsonian translocations and a pericentric inversion between *T. inunguis* (2n = 56) and *T. manatus* (2n = 48) reveals a high rate of chromosomal changes within the genus *Trichechus*, between 1 to 5 changes/myr.

The analysis of the *Cyt b* gene by Vianna et al. [15] suggested that *T. inunguis* might belong to an older lineage of manatees adapted to freshwater. Therefore, the species may have a more conserved gene sequence than *T. manatus* and *T. senegalensis*. The new insights of De Souza et al. [16] on the phylogenetic relationship of *T. manatus* and *T. inunguis* provide more specific answers about the differences between these species, which were also reinforced in the present study. The chromosomal changes in APK that led to the karyotype of *T. manatus* and *T. inunguis* range from 7 to 4 changes, respectively; this indicates that *T. inunguis* shares a more conserved karyotype with APK, while *T. manatus* presents apomorphies that show a condition that is more derived from APK. Notably, the chromosomal evolution of the *Trichechus* genus will be elucidated only after the application of TML probes to *T. senegalensis*.

## Conclusion

Here, we evaluated by chromosome painting important data on the karyotypic differences between the species *Trichechus manatus* and *Trichechus inunguis* and the phylogenetic relationships of these species to other representatives of Paenungulata. The high rate of chromosomal changes in manatees shows them as outliers of the Afrotheria clade. Despite this, the homeologies between the paenungulate karyotypes are still very conserved, with evidence even in the G-banding pattern. The shared HSA syntenies in *T. inunguis* reveal it as a representative of the placental mammalian taxons Afrotheria and Paenungulata. The phylogenetic signals found in *T. inunguis* show that the species shares more conserved chromosomal signals with the ancestral karyotype of Paenungulata (APK) compared to hyrax (*Procavia capensis*), the African elephant (*Loxodonta africana*), and Florida manatee (*Trichechus manatus latirostris*). From a phylogenetic perspective, the karyotype of *T. m. latirostris* is the most derived among the representatives of Paenungulata. Furthermore, the data from this study also point to the phylogenetic position between *T. manatus* and *T. inunguis*, showing that *T. manatus* presents a more recent condition than *T. inunguis* among the American *Trichechus*. However, complete understanding of the chromosomal evolution of the genus will be possible only after chromosomal painting of *T. senegalensis*.

## Methods

Blood samples were collected from a male and a female of *Trichechus inunguis* under the SISBIO license number (Number: 44915–1). Chromosomal preparations were obtained from temporary lymphocyte cultures. Cultivation was performed in RPMI 1640 medium (Vitrocell) with fetal bovine serum (FBS) and phytohemagglutinin and incubated at 37ºC in 5% CO_2_ for 96 h. Metaphases were analyzed according to chromosome morphology and organized karyotype according to Assis et al. [19]. The G-banding pattern was performed using Seabright’s protocols [43], the best G banded karyotype was published for us in Noronha et al. [22]. The whole chromosome probes used in this study were described by Pardini et al. [2], where 23 peaks were generated from a male of *Trichechus manatus latirostris* (TML; 2n = 48) by flow-sorted, with 17 peaks of a single chromosome (TML 1, 5, 8, 9, 10, 11, 12, 14, 15, 16, 17, 18, 20, 20, 22, 23, Y) and 3 peaks composed of two chromosomes (2 + 4, 3 + 7 and 6 + X). The TML 20 chromosome is present in 2 separate peaks, possibly due to the heterochromatin difference between homologs carrying the nucleolus organizer region (NOR) and presenting nonspecific markings on the chromosomes. TML chromosomes 11, 14, and 17 have both peaks in their pure form and also mixed peaks with other chromosomes, such as 11 + 13, 14 + 19, and 17 + 21, making it possible to characterize the TML chromosome 19 in hybridizations (Table 1).

In situ hybridizations were performed according to Yang and Graphodatsky [44], photographed with a Zeiss Axiocam camera, coupled to a Zeiss microscope, and analyzed with AxioVision Rel software. 4.6. The analyzes followed the interpretation of the presence/absence of signals in the chromosomes; comparative idiograms were set up in Photoshop CS6 software for cytogenetic analysis between the investigated species.

## Data Availability

All data generated or analyzed during this study are included in this published article.

## Abbreviations

2n: Diploid number
APK: Ancestral Paenungulata Karyotype
CO_2_: Carbon Dioxide
*Cyt* b: Cytochrome B
D-loop: Displacement loop
AEK: Ancestral Eutherian Karyotype
FBS: Fetal bovine serum
FISH: Fluorescence In-Situ Hybridization
FN: Fundamental number
HSA: *Homo sapiens*
LAF: *Loxodonta africana*
NOR: Nucleolar organizer regions
OAF: *Orycteropus afer*
PCA: *Procavia capensis*
SISBIO: Sistema de Autorização e Informação em Biodiversidade
TIN: *Trichechus inunguis*
TMA: *Trichechus manatus*
TML: *Trichechus manatus latirostris*
